# The Ambiguous Role of *NKX2-5* Mutations in Thyroid Dysgenesis

**DOI:** 10.1371/journal.pone.0052685

**Published:** 2012-12-28

**Authors:** Klaartje van Engelen, Mathilda T. M. Mommersteeg, Marieke J. H. Baars, Jan Lam, Aho Ilgun, A. S. Paul van Trotsenburg, Anne M. J. B. Smets, Vincent M. Christoffels, Barbara J. M. Mulder, Alex V. Postma

**Affiliations:** 1 Department of Cardiology, Academic Medical Center, Amsterdam, The Netherlands; 2 Department of Clinical Genetics, Academic Medical Center, Amsterdam, The Netherlands; 3 Interuniversity Cardiology Institute of The Netherlands (ICIN), Utrecht, The Netherlands; 4 Heart Failure Research Centre, Department of Anatomy and Embryology, Academic Medical Center, Amsterdam, The Netherlands; 5 Department of Pediatric Cardiology, Academic Medical Center, Amsterdam, The Netherlands; 6 Department of Pediatric Endocrinology, Academic Medical Center, Amsterdam, The Netherlands; 7 Department of Radiology, Academic Medical Center, Amsterdam, The Netherlands; Cardiff University, United Kingdom

## Abstract

*NKX2-5* is a homeodomain-containing transcription factor implied in both heart and thyroid development. Numerous mutations in *NKX2-5* have been reported in individuals with congenital heart disease (CHD), but recently a select few have been associated with thyroid dysgenesis, among which the p.A119S variation. We sequenced *NKX2-5* in 303 sporadic CHD patients and 38 families with at least two individuals with CHD. The p.A119S variation was identified in two unrelated patients: one was found in the proband of a family with four affected individuals with CHD and the other in a sporadic CHD patient. Clinical evaluation of heart and thyroid showed that the mutation did not segregate with CHD in the familial case, nor did any of the seven mutation carriers have thyroid abnormalities. We tested the functional consequences of the p.A119S variation in a cellular context by performing transactivation assays with promoters relevant for both heart and thyroid development in rat heart derived H10 cells and HELA cells. There was no difference between wildtype NKX2-5 and p.A119S NKX2-5 in activation of the investigated promoters in both cell lines. Additionally, we reviewed the current literature on the topic, showing that there is no clear evidence for a major pathogenic role of *NKX2-5* mutations in thyroid dysgenesis. In conclusion, our study demonstrates that p.A119S does not cause CHD or TD and that it is a rare variation that behaves equal to wildtype NKX2-5. Furthermore, given the wealth of published evidence, we suggest that *NKX2-5* mutations do not play a major pathogenic role in thyroid dysgenesis, and that genetic testing of *NKX2-5* in TD is not warranted.

## Introduction

Persistent congenital hypothyroidism of thyroidal origin is a relatively common disorder, occurring in about 1/2500 live births [1]. In 85% of cases it is caused by thyroid dysgenesis (TD), consisting of agenesis, hypoplasia or ectopia of the thyroid gland [2]. TD is a heterogeneous disorder that occurs mostly sporadically, though 2% of cases are reported as familial [3]. The pathogenesis of TD is largely unknown; possible roles for environmental, genetic and epigenetic factors have been suggested, and in a minority of humans with TD mutations in *NKX2-1* [4], *FOXE1* [5], *PAX8* [6] and *TSHR* [7] have been identified. Additionally, in a recent study mutations in *NKX2-5* were reported in a small proportion of patients with persistent congenital hypothyroidism [8].

*NKX2-5* encodes a homeodomain-containing transcription factor that is expressed during thyroid development (for review see [9]), but it is mainly known to play a crucial role in heart development [10]. *NKX2-5* mutations have been found in a subset of patients with congenital heart disease (CHD), mostly septal defects [11], [12]. As CHD is overrepresented among children with TD and vice versa, a developmental association between the cardiac and thyroid systems has been suggested [13]–[15].

We screened families and individual patients with CHD for mutations in *NKX2-5*. In this paper we focus on the p.A119S variation, which we found in two probands. Dentice *et al.* reported this as a causative mutation in a child with ectopic thyroid gland, with functional studies showing a dominant negative effect of the mutation [8]. We evaluated the heart and the thyroid gland in our two families with a total of seven p.A119S carriers and we performed follow-up functional studies. Additionally, we discuss existing literature on the connection between *NKX2-5* mutations and TD.

## Methods

### Ethics Statement

This study was approved by the Medical Ethical Committee of the Academic Medical Center in Amsterdam. Written informed consent was obtained from all participants.

### Patients and Clinical Evaluation

DNA from 303 patients with primum atrial septal defect (ASDI, n = 271) or secundum atrial septal defect (ASDII, n = 32) was extracted from CONCOR, a nationwide registry and DNA bank for adult patients with CHD, described in detail elsewhere [16]. Additionally, probands of 38 families with multiple (at least two) affected patients with several forms of CHD, identified at the departments of clinical genetics or cardiology of the AMC, were included. In this study, we focused on patients who were found to carry the p.A119S NKX2-5 variation. Probands with this variation, as well as their available family members, were clinically evaluated. Medical records were analyzed and all individuals underwent physical examination with attention to syndromic features. Cardiologic examination consisted of a 12-lead electrocardiogram (ECG) and two-dimensional echocardiography, which were assessed by a cardiologist who was blinded for the mutational status. Thyroid ultrasound and thyroid function analysis were used to investigate the thyroid gland. TSH and free T4 were measured by time-resolved fluoroimmunoassay (Delfia, hTSH Delfia Ulta resp. FT4 Delfia, Perkin Elmer, Turku, Finland), detection limits: 0.01 mU/L for TSH and 2 pmol/L for free T4, total assay variation: 4–5% for TSH and 6–7% for free T4. Thyreoglobulin and anti-TPO were measured by chemiluminescence immunoassay (LUMI-test Tg resp. anti-TPO, BRAHMS, Berlin, Germany), detection limits: 1 pmol/L for Tg and 30 kU/L for anti-TPO, total assay variation: 7–13% for Tg and 8–12% for anti-TPO. The results of the ultrasound were analyzed by a radiologist who was blinded for mutational status as well as cardiologic status.

### Mutation Analysis

Genomic DNA of CHD patients as well as relatives of patients carrying the A119S variation was extracted from peripheral blood according to standard procedures. Coding regions and intron–exon boundaries of NKX2-5 (NM_004387.3) were analyzed using direct sequence analysis on an ABI3730xl capillary sequencer using Big-Dye Terminator v3.1 (Applied Biosystems). Data were analyzed using Codoncode analysis software (v3.1, CodonCode Corporation). In the proband with the p.A119S variation who had aortic coarctation and bicuspid aortic valve, sequence analysis of the NOTCH1 gene was also performed.

### Plasmid Constructs and Transfections

Human clones for NKX2-5 and TBX5 were obtained from the IMAGE consortium [17]. The human clones were in the following vectors: pCMVSport6-hNKX2-5 and pcDNA3.1-hTBX5. Promoter construct for ANF-luc is as described before [18], the promoters for Dio2, Tg and TPO were cloned from their appropriate species, as described before [8], and subcloned into pGL3 basic expression vectors (Promega). Expression and promoter constructs were all sequence verified. pCMVSport6-hNKX2-5 mutants (p.A119S, p.N188K) were constructed using site-directed mutagenesis (Strategene). Transfections were performed using polyethylenimine (25 kDa, linear, Brunschwick).

### EMSA, Probe Annealing

Radioactive Electrophoretic Mobility Shift Assay (EMSA) was performed using the following wildtype sequence as probes: 5′-TCTGCTCTTCTCACACCT**TTGAAGTGGGG**GCCTCTT and its complementary oligo (5'-GCCTCAAGAGGC**CCCCACTTCAA**AGGTGTG), as described before [18], The specific conditions were as follows: bandshift buffer (BB) (10 mM Tris pH 7.9, 10% glycerol, 50 mM NaCl, 0.5 mM EDTA); non-specific competitor Spermidine 3-HCl (Sigma, S2501) at a concentration of 1 µg/µl;prepared according manufacturers instruction. First 5.0 µg crude nuclear cell extracts were pre-incubated for 5 min at +15 to +25°C in a reaction containing 14 µl BB, 1 µl of the non-specific competitor spermidine, 1 µg BSA, 1 mM DTT and supplemented with H20 up to 20 µl. Input was corrected for Nkx2.5 expression and total amount protein was kept constant at 5.0 µg by addition of empty vector nuclear extracts. Then 2 µl of labelled Nkx-specific probe (30000 c.p.m) was added. Complexes were allowed to form for 20–25 min at +15 to +25°C. The samples were loaded on 6%-TBE polyacrylamide gel which was prerunned at RT for 30 min at 25 V. Complexes were separated at 4 V/cm at RT for 60 min. Gels were dried unfixed on Whatman 3 MM and exposed for autoradiography.

### Luciferase Assay

Neonatal rat heart myocytes, immortalized with a temperature-sensitive SV40 antigen (H10 cells [19], were grown in standard 12-wells plates in DMEM supplemented with 10% FCS (Gibco-BRL) and glutamine. HeLa cells were grown according to standard culturing conditions [20]. 700 ng Nppa/TPO/Dio2/Tg-luciferase constructs were co-transfected with 1 ng of cmv-renilla vector, as normalization control (Promega), together with appropriate combinations of expression constructs (pCMVSport6-hNKX2-5, pcDNA3.1-hTBX5) up to 900 ng. Measurements were performed on a Glomax 20/20 luminometer. Triplo transfection experiments were repeated at least three times for each condition, data were corrected for intersession variation as described [21]. Statistical analysis was performed using two-tailed t-test, P<0.05 was considered significant.

### Nuclear Localization

Cos7 cells [22] were seeded in standard 12-wells plates and transfected with 500 ng WT or p.A119S NKX2.5. 24 h post-transfection, cells were fixed in 2% paraformaldehyde, permeabilized using 0.3% Triton X-100, and incubated with rabbit anti-Nkx2-5 (Santra cruz) and DAPI (Sigma).

## Results

### Mutational Analysis

We identified a total of three missense NKX2-5 variations in our cohort of 341 CHD patients: a p.C270Y variant in a patient with ASDI and cleft mitral valve, and twice the p.A119S variant in separate probands (see below). We will only discuss the results of the p.A119S variant, as the other variation is outside the scope of this study.

**Figure 1:**
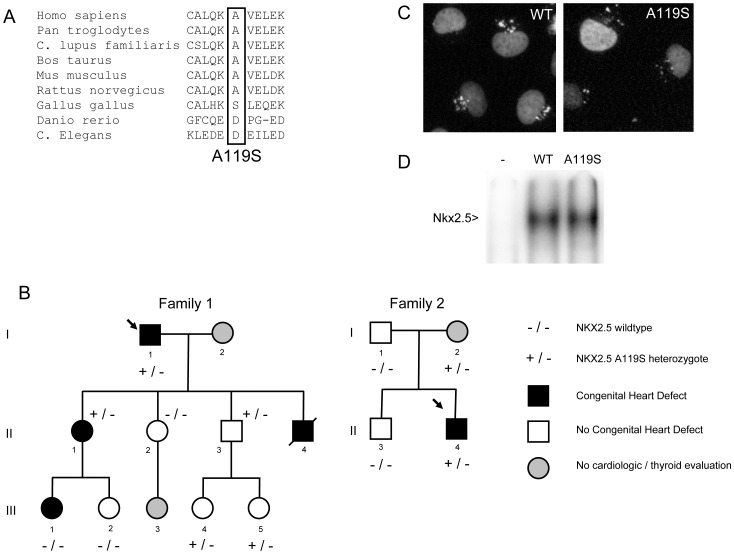
Family pedigrees, aminoacid alignments, nuclear localization of NKX2-5 protein and electro mobility shift assay. A. Pedigrees of the two families with the p.A119S NKX2-5 mutation. Individuals with congenital heart defects are indicated with a filled black symbol, while individuals with normal echocardiography are indicated with a white symbol. Grey symbols represent individuals that have not been evaluated clinically. A slash denotes a deceased individual; the proband is indicated by an arrow. None of the evaluated family members showed thyroid abnormalities. Heterozygous carriers of p.A119S are represented by +/− and non-carriers by −/−. B. Multiple alignments of aminoacids of the region surrounding p.A119 for various species. C. Nuclear localization of either wildtype or p.A119S NKX2-5 protein in COS7 cells. Nuclei are stained in green, red represents either the wildtype or mutant protein, orange indicates nuclei that are positive for wildtype or mutant protein. D. Electro mobility shift assay in cos7 cells, using the published nkx2.5 binding site [26]. Wildtype nkx2.5 protein and p.A119S Nkx2.5 protein bind equally well, – is untransfected control.

The nucleotide change from G to T at codon 119 in *NKX2-5* was identified in two patients. This results in the substitution of an alanine for a serine leading to p.A119S. This variation was present once in a proband from one of the 38 families tested and once in a proband from 303 sporadic patients with ASD. The p.A119S variation was not found in 200 local controls. Data from the NHLBI exome sequencing project shows that it is a very rare variation with a minor allele frequency of 0.001% (7/6470 individuals, rs1378526) [23]. Alignment of the aminoacids of proteins of different species shows that this position is conserved up to rat Nkx2-5 (Figure 1A). However, chicken Nkx2-5 protein actually has a serine at this position, though the surrounding aminoacids differ. The pedigrees of the families with the p.A119S variation are shown in Figure 1B. Table 1 summarizes the clinical features of both families. Analysis of NOTCH1 in the proband of family 1, who had aortic coarctation and bicuspid aortic valve, did not show a pathogenic mutation.

**Table 1:** Clinical details of the family members.

Patients		A119S	Heart		Thyroid								Remarks
	Sex/age at evaluation (years)		CHD	ECG	Position in neck	Aspect	Volume left lobe (ml)	Volume right lobe (ml)	TSH (mE/L)	Free T4 (pmol/L)	Thyreo-globulin (pmol/L)	Anti-TPO antibodies	
**Family 1**													
I-1	M/81	+	CoA, BAV	Atrial fibrillation	Normal	Homogenous	9.5	9.0	2.20	14.5	4	Neg	
II-1	F/51	+	VSD	Normal	Normal	Homogenous	4.6	3.3	3.60	12.7	9	Neg	
II-2	F/49	−	None	Low voltages	Normal	Homogenous	3.7	3.9	1.60	12.3	5	Neg	
II-3	M/48	+	None	Normal	Normal	Homogenous	5.0	7.5	2.10	14.4	6	ND	
II-4	M/10 days	ND	CoA	ND	ND	ND	ND	ND	ND	ND	ND	ND	Deceased at age 10 days
III-1	F/24	−	VSD	Rigth axis deviation	Normal	Homogenous	2,9	3	1.70	13.6	9	Neg	
III-2	F/21	−	None	Normal	Normal	Homogenous	4.6	4.8	1.50	13.4	6	Neg	
III-4	F/20	+	None	Normal	Normal	Homogenous	3.0	6.0	1.40	15.3	6	Neg	
III-5	F/12	+	None	Normal	Normal	Homogenous	2.6	3.5	ND	ND	ND	ND	
**Family 2**													
I-1	M/60	−	None	ND	Normal	Homogenous	4.4	5.9	1.80	16.2	4	Neg	
I-2	F/62	+	None reported	ND	ND	ND	ND	ND	ND	ND	ND	ND	
II-1	M/35	−	None	ND	Normal	Homogenous	6.7	8.9	1.50	14.2	6	Neg	
II-2	M/31	+	ASDI, ASDII	RBBB	Normal	Homogenous	5.6	5.0	1.40	17.0	2	Pos(620 kU/l)	Homogenous solid nodule in left thyroid lobe, diameter 6 mm

ND, not determined; neg, negative; pos, positive; CHD, congenital heart disease; CoA, aortic coarctation; BAV, bicuspid aortic valve; VSD, ventricular septal defect; ASDI, ostium primum atrial septal defect; ASDII, ostium secundum atrial septal defect; RBBB, right bundle branch block.Anti-TPO antibodies ‘negative’ means value <50 kU/l.

### Family Phenotypes

#### Family 1

The p.A119S variation was found in the proband (I-1), who had an aortic coarctation and bicuspid aortic valve diagnosed at age 45 years. The coarctation was surgically corrected at age 45 years and an artificial aortic valve was implanted at age 73 years because of severe calcification and stenosis. The probands’ youngest son (II-4) died 20 days after birth, post-mortem pathology revealing aortic coarctation. The oldest daughter of the proband (II-1) was born with a ventricular septal defect (VSD) that closed spontaneously during childhood. Her oldest daughter (III-1) also had a VSD which was surgically closed when she was 7 months old. Echocardiography was normal in the other family members. Normal location, volume and structure of the thyroid gland were shown by ultrasound in all investigated family members. Thyroid function was also normal in all individuals. The p.A119S variation did not segregate with the cardiac defects within the family, as III-1 is affected but she does not have the mutation and several family members without any evidence of CHD carried the mutation (II-3, III-4, III-5).

#### Family 2

The p.A119S variation was also found in a sporadic patient with ASDI with cleft mitral valve as well as small ASDII, for which a surgical correction took place at the age of 5 years. Thyroid ultrasound showed normal location and volume of the gland, but a small nodule was present in the left lobe. Additionally, anti-TPO antibodies were positive (620 kU/l) with normal thyroid function tests. These abnormalities are frequent in the general population [24], [25], and we therefore do not consider them to fall outside the range of expected findings. The probands’ mother was found to carry the p.A119S variation and the father and brother did not. The mother was not available for clinical evaluation, though she did not have a history of cardiac or thyroid disorders. The probands’ father had a myocardial infarction at age 55. He did not have CHD. Echocardiography in the probands’ brother did not show CHD either. Thyroid evaluation of the probands’ father and brother was also normal.

### Normal Sub Cellular Distribution of the NKX2-5 p.A119S Protein

To be able to regulate transcription and exert its function, the NKX2-5 protein needs to be present in the nucleus. The localization of the p.A119S NKX2-5 protein was assessed by transfecting it into rat heart-derived cells (H10) and COS7 cells. The localization of mutant and WT proteins was visualized with an antibody against Nkx2-5. Figure 1C shows that both the wildtype and p.A119S NKX2-5 protein localize exclusively inside the nucleus, indicating that the process of nuclear import is not affected by the variation.

### No Functional Defect in DNA Binding of p.A119S Nkx2.5

The transcription factor Nkx2.5 activates its target genes by binding to the DNA. To test whether the DNA binding capacity of p.A119S Nkx2.5 was altered, we used an electrophoretic mobility shift assay (EMSA) [18]. A fragment of the *Nppa* promoter was used, a well characterized promoter relevant in heart development, containing a functional Nkx2.5 binding element [26]. As shown in figure 1D, both wildtype and p.A119S Nkx2.5 protein bind equally well, indicating that there is no difference in DNA binding capacity between p.A119S and wildtype Nkx2.5 protein.

### No Difference in Promoter Activations of Wildtype NKX2-5 or p.A119S NKX2-5

To test the functional consequences of the p.A119S variation in a relevant cellular context, we used reporter assays in which the proximal *NPPA* promoter (−270 to +1) [26], and the *Dio2*, *Tg* and *TPO* [8] promoters involved in thyroid gland function, were fused to a luciferase reporter. These promoters all contain functional binding sites for NKX2-5. We also tested NKX2-5 in combination with the TBX5 transcription factor as TBX5 synergizes with NKX2-5 in the activation of the *NPPA* promoter [26]. These transactivation assays were performed in both H10 cells and HELA cells, as used in the original publication of Dentice *et al.* on the possible connection between p.A119S and TD [8]. As a negative control we also used the p.N188K NKX2-5 mutant, reported as causative in a family with five affected presenting with atrial septal defects, Ebsteins anomaly and abnormal AV conduction [27]. No thyroid abnormalities were reported for this mutation. p.N188K introduces a mutation in the homedomain of Nkx2.5, an element conserved in all members of the Nkx protein family and known to directly contact adenine in the major groove of DNA. The p.N188K mutation leads to a complete loss-of-function in DNA binding [28] and can therefore serve as a negative control.

**Figure 2:**
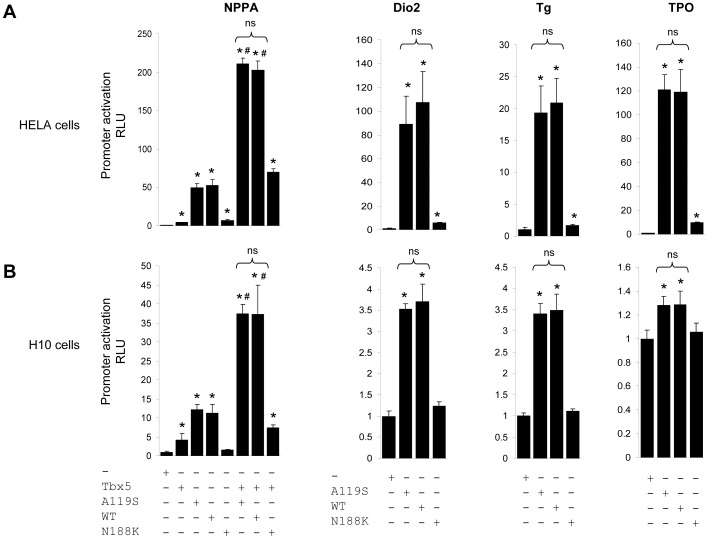
Relative activation of the Nppa, Dio2, Tg and TPO promoters in combination with wildtype NKX2-5, p.A119S NKX2-5, p.N188K NKX2-5 or TBX5. A. In H10 cells. B. In HELA cells. Significant differences between vector and condition tested are marked with *, P<0.05. # denotes a significant difference between conditions tested with and without TBX5, P<0.05. Error bars represent standard deviation (SD). Each condition has been tested at least in three independent triplicate experiments.

In the H10 cells, the wildtype NKX2-5 and the p.A119S protein both significantly activated the *NPPA* promoter driven reporter. When transfected together with TBX5 both wildtype NKX2-5 and p.A119S NKX2-5 also activated the reporter construct synergistically (Figure 2A). There was no difference between wildtype NKX2-5 and p.A119S NKX2-5 in the activation of the *NPPA* promoter construct for any condition tested. Likewise, we observed no difference in activation of the *Dio2*, *Tg* or *TPO* promoter constructs between wildtype and p.A119S in H10 cells. We repeated all experiments in HELA cells, and found stronger activation of all constructs in comparison to the H10 cells. However, once again, we observed no difference in activation of any promoter tested between wildtype NKX2-5 and p.A119S NKX2-5 (Figure 2B).

## Discussion

In our population of 303 patients with ASD and 38 probands from families with CHD, we found the NKX2-5 p.A119S variation in two patients. The variation did not segregate with CHD in the familial case, nor were any signs of TD present in the seven mutation carriers. Furthermore, functional studies showed no difference between wildtype and p.A119S protein in activation of four different promoters in either H10 cells or HELA cells. Taken together, our results strongly suggest that the p.A119S variation behaves similar to wild type NKX2-5 and that it has no discernible pathogenic role in either CHD or TD.

NKX2-5 belongs to the NK-2 family of homeodomain-containing transcription factors, which are conserved from flies to humans [10]. Its role as a transcription regulator during early embryonic heart developmental has been known for many years, and mutations in NKX2-5 are found in patients with CHD [10]–[12]. NKX2-5 has also been shown to be required for thyroid development in animal studies [8], [29]. The link between thyroid development and NKX2-5 was highlighted by a recent publication of Dentice *et al.* [8], who reported three variations in NKX2.5 in four of the 241 patients with persistent congenital hypothyroidism studied, amongst them the p.A119S variation. They performed functional studies and showed a reduced DNA binding capacity and reduced transactivation properties with a dominant negative effect for p.A119S in comparison to wildtype Nkx2-5. The p.A119S mutation identified by Dentice *et al.* occurred in a girl with an ectopic thyroid gland. Her mother, who also carried the mutation, had auto-immune hypothyroidism but no evidence of TD and both were without evidence of CHD. Our molecular testing of the p.A119S variation in both rat heart derived (H10) cells and HELA cells showed no difference in transactivation of any of the four promoters tested (*NPPA*, *Tg*, *Dio2*, *TPO*), which is in contrast to the results obtained by Dentice *et al.* Nevertheless, the results of our functional studies are in agreement with our clinical data as the mutation did not segregate with CHD in our familial case and none of our seven mutation carriers had thyroid disease. Moreover, the A119S variation is present in the general population at a low rate (0.001%) and classified as a SNP [23]. In general, we conclude that we cannot uphold the results obtained by Dentice *et al.*, and it is unclear why the molecular results between the two studies are different, as the same proteins, promoters and cell lines were used. One difference is the fact that we did include a negative control (p.N188K) to show that our assay is robust and no confounding variables acted on the experiment.

Given the above, an important question is to what extent NKX2-5 mutations are involved in the pathogenesis of TD. In addition to p.A119S, three other NKX2-5 variations have been reported in literature thus far to be associated with TD: p.R25C, p.S265R and p.R161P [8], [30]. The p.R25C variation has been identified in several patients with CHD, none of whom were reported to have TD [31]. Moreover, this variation is present in 1% of the general population as a SNP (rs2893667) [23], making it unlikely that it plays any pathogenic role in TD. In contrast, both the p.S265R and the p.R161P variation have not been reported in the general population. The p.S265R variation was reported in a girl with TD who also carried a mutation in the PAX8 promoter region [30] and the mutant protein was shown to have a reduced function. However, as the girls’ healthy brother, father and grandmother also carried the NKX2-5 variation and the PAX8 mutation may have accounted for TD in the girl, there is no direct evidence that the p.S265R variation causes TD. The p.R161P NKX2-5 variation was found in a TD patient; however her father also carried the mutation but had no TD or CHD. Taken together, none of the four currently published NKX2-5 variations have been demonstrated to segregate with a phenotype of TD within a family. Although incomplete penetrance cannot be totally excluded, there is no strong genetic evidence of a clear pathogenic effect of the mutations.

To gain further insight into the role of NKX2-5 mutations in TD, a cohort of TD patients can be investigated for mutations in NKX2-5. However, the NKX2-5 gene has been analyzed in over 460 congenital hypothyroidism patients to date, but no additional mutations were identified (Table 2) [14], [15], [32]–[35]. Interestingly, 51 of these patients also had CHD [14], [33]. Furthermore, none of the more than 150 CHD patients with a demonstrated NKX2-5 mutation [12] were reported to have thyroid problems.

**Table 2:** Studies analyzing *NKX2-5* in patients with persistent congenital hypothyroidism.

Author	Type of patients	N ofpatients	N of mutation carriers (%)	Remarks
Dentice et al., 2006 [8]	Persistent CH (athyreosis 53; thyroid ectopy 98; thyroid hypoplasia 15; 75CH without goiter)	241	4 (1,7)	Two mutations (p.A119S and p.R25C), present in 3/4 patients, have been reported as a SNP [23].
Al Taji et al., 2007 [33]	Persistent primary non-auto-immune, non-goitre hypothyroidism AND CHD	15	0 (0)	
Ramos et al., 2009 [15]	Thyroid hypoplasia or athyreosis	35	0 (0)	
Cangul et al., 2009 [32]	Primary non-auto-immune, non-goitre hypothyroidism, from consanguineous families	9	0 (0)	In an additional 130 patients from consanguineous families linkage to the NKX2.5 locus was assumed to be excluded because heterozygosity for the gene was detected (no mutational analysis performed).
Narumi et al., 2010 [35]	Permanent primary CH diagnosed by neonatal screening (thyroid ectopy 37; thyroid aplasia 6; thyroid hypoplasia 8; other 51)	102	0 (0)	
Passeri et al., 2011 [14]	CHD and non-autoimmune CH (normal thyroid volume 35; hemiagenesis 1)	36	0 (0)	
Hermanns et al., 2011 [30]	nm	nm	1	Case report: the p.S265R variation was identified in a girl with thyroid dysgenesis who also carried a mutation in the PAX8 promoter region.
Brust et al., 2012 [34]	Thyroid dysgenesis (thyroid ectopy 13; hypoplasia 11; athyreosis 3)	27	0 (0)	

CHD, Congenital Heart Disease; CH, Congenital Hypothyroidism; nm, not mentioned.

Although there is a lack of evidence for a strong pathogenic effect of NKX2-5 mutations in human TD, Nkx2-5 has been shown to be involved in thyroid development. Evidence for this comes from studies using wildtype Nkx2-5 mice, showing Nkx2-5 expression in the thyroid primordium up to E11.5 [8]. Moreover, Nkx2-5 knockout mice demonstrate thyroid bud hypoplasia [8], [28]. Although these studies suggest that absence of Nkx2-5 could lead to (a form of) TD, one should keep in mind that these observations are based on Nkx2-5 null mice, which die around E9-10 [36]. In contrast, heterozygous knockout mice are viable and are not reported to have TD [37]. This suggests that the loss of one Nkx2-5 allele is tolerated, perhaps by compensation during development by paralogue genes such as NKX2-1, which activates the same promoter regions as NKX2-5.

Altogether, given the wealth of published evidence, we believe that NKX2-5 mutations do not play a major pathogenic role in TD. A role of NKX2-5 as a genetic modifier cannot entirely be excluded though. To our opinion, there is currently not enough evidence to warrant routine genetic testing for NKX2-5 mutations in TD patients, and vice versa, to evaluate the thyroid in individuals carrying an NKX2-5 mutation.

In conclusion, the results of our study demonstrate that p.A119S does not cause CHD or TD and that it is a rare variation that behaves equal to wildtype NKX2-5. Furthermore, given the lack of clear evidence of pathogenicity of the reported NKX2-5 mutations, the high amounts of patients with TD without an NKX2-5 mutation and the absence of TD in NKX2-5 mutation carriers, we suggest that NKX2-5 mutations do not play a major pathogenic role in thyroid dysgenesis and that genetic testing for NKX2-5 in TD is not warranted. A role of NKX2-5 as a genetic modifier cannot entirely be excluded.
